# Progress of phototherapy for osteosarcoma and application prospect of blue light photobiomodulation therapy

**DOI:** 10.3389/fonc.2022.1022973

**Published:** 2022-10-13

**Authors:** Jiali Yang, Qiqi Fu, Hui Jiang, Yinghua Li, Muqing Liu

**Affiliations:** ^1^ School of Information Science and Technology, Fudan University, Shanghai, China; ^2^ Academy for Engineering and Technology, Fudan University, Shanghai, China; ^3^ Shanghai Fifth People’s Hospital, Fudan University, Shanghai, China; ^4^ Zhongshan Fudan Joint Innovation Center, Zhongshan, China

**Keywords:** osteosarcoma, phototherapy, photobiomodulation therapy, cellular mechanism, blue light

## Abstract

Osteosarcoma (OS) is the most common primary malignant bone tumor that mainly affects the pediatric and adolescent population; limb salvage treatment has become one of the most concerned and expected outcomes of OS patients recently. Phototherapy (PT), as a novel, non-invasive, and efficient antitumor therapeutic approach including photodynamic therapy (PDT), photothermal therapy (PTT), and photobiomodulation therapy (PBMT), has been widely applied in superficial skin tumor research and clinical treatment. OS is the typical deep tumor, and its phototherapy research faces great limitations and challenges. Surprisingly, pulse mode LED light can effectively improve tissue penetration and reduce skin damage caused by high light intensity and has great application potential in deep tumor research. In this review, we discussed the research progress and related molecular mechanisms of phototherapy in the treatment of OS, mainly summarized the status quo of blue light PBMT in the scientific research and clinical applications of tumor treatment, and outlooked the application prospect of pulsed blue LED light in the treatment of OS, so as to further improve clinical survival rate and prognosis of OS treatment and explore corresponding cellular mechanisms.

## Introduction

Osteosarcoma (OS) is the high-grade malignant bone tumor, mainly occurring in children and adolescents but a second and smaller peak in elderly patients (1). It exhibits a preference to arise in the metaphysis of long bones and the sites of rapid bone growth, such as the distal femur (43%), proximal tibia (23%), proximal humerus (10%), skull, mandible, and other sites (2, 3). Despite the high survival rate of OS (70%∼80%) with traditional therapy nowadays, around 20% after metastasis is still dismal (3, 4). In particular, limb salvage, postoperative quality of life, and inhibition of metastasis are the most important issues in the current treatment of OS (3, 5). Therefore, the exploration of new treatment methods is extremely important.

At present, some new antitumor methods, such as targeted therapy and immunotherapy, continue to play an important role in the treatment of a variety of cancers (6, 7). However, due to the defects of drug resistance and specific treatment, there are still many problems in clinical treatment. On the other hand, phototherapy, a novel, non-invasive, and efficient antitumor therapy, including photodynamic therapy (PDT), photothermal therapy (PTT), and photobiomodulation therapy (PBMT), has gradually become a hot spot in the current scientific research and clinical experiments of OS (8). Currently, the laser and LED are two common light sources in phototherapy. LED devices are gradually widely applied in cancer treatment research fields because of their cheapness, large radiation range, and wearability (9). Therefore, LED phototherapy will develop rapidly in deep cancer treatment in the future.

Current research shows that continuous wave light PBMTs have been gradually applicated in dermatology (acne, eczema, and psoriasis) and skin cancer *in vitro* and *in vivo*. For example, Oh et al. (2015) found that blue light had antiproliferative and proapoptotic effects on melanoma cells and reduced tumor growth in mouses (10). Chen et al. (2021) summarized the research status of light therapy for melanoma in the past 20 years and also found that blue light can effectively inhibit the proliferation of melanoma cells (11). Besides, the growth of malignant glioma (12), mouse B-cell lymphoma (13), colon cancer (14), and pancreatic cancer (15) can be effectively inhibited by blue light in animal experiments. However, due to the limited tissue penetration of continuous wave light, there are still limitations in the study of deeper tumors. On the other hand, pulse mode LED light can make up for the defect of low tissue penetration of continuous wave light due to high peak irradiance (16, 17). At the same time, adjusting the pulse mode can effectively ensure the stability of temperature, so as to achieve the inhibitory effect of high light intensity on superficial epidermal tumors such as skin tumors (11). Although some studies have shown that phototherapy can effectively inhibit tumor proliferation *in vivo* and *in vitro*, numerous studies have not carried out long-term safety testing, so the safer spectral application mode and subsequent safety testing related research need to be further explored.

In brief, we described the development and current treatments for OS before focusing on research progress from traditional therapies to new therapeutic approaches including targeted therapy, immunotherapy, PTT, PDT, and PBMT that played an irreplaceable role in improving survival rate of clinical for this disease. Finally, we mainly focused on the research status of PBMT of blue LED light in the treatment of OS, looked ahead to the results of existing research in this laboratory, and further discussed the research prospect of blue light and the related mechanism of phototherapy in OS.

## Occurrence and metastasis of osteosarcoma

OS is a genetically diverse and karyotypically complex cancer characterized by chromosomal instability, copy number alterations, and chromothripsis, mainly originated from bone with high concentrations of mesenchymal progenitor cells and developed in bone (18, 19). A number of studies identify mesenchymal stem cells (MSCs) or committed osteoblast precursors as the OS cell of origin (20). As research on OS deepens, more etiologies are identified such as several chemical agents (beryllium, methylcholanthrene), viruses (FBJ), and hereditary diseases (Li–Fraumeni syndrome, Rothmund–Thomson syndrome, and Bloom syndrome), while leading to prevalent alterations in tumor-suppressor genes, including *TP53* (>90%), *RB1* (29%), *DLG2* (53%), and *ATRX* (29%) (21, 22). As mentioned earlier, the tumor-suppressor pathway regulated by *p53* and *Rb* is one of the main factors involved in the etiopathogenesis of OS, mainly regulated in DNA damage and cell-cycle progress (23). Chou and Gorlick (2006) provided a paradigm in the potential pathogenesis of OS, mainly including the main development mechanisms of precursor cells and early OS cells (19, 24).

Recent findings also indicate that OS progression is a multistep and complicated process associated with an intricate tumor microenvironment. The mesenchymal stroma played a critical role in OS growth, maintenance, and micrometastasis, and the interactions between OS cells and multiple factors (cytokines, exosomes, metabolites) in mesenchymal stroma reveal the complexity of OS occurrence (19).

OS cells perform a high propensity to spread and metastasize, which seems to be the most important internal factor for poor prognosis in OS patients (2). The inability to detect the occurrence of OS metastasis in a timely and rapid manner is the main reason for the low cure rate of metastatic patients. Therefore, new detection methods and comprehensive and in-depth understanding of the molecular mechanisms of OS metastasis are urgently needed to develop. Studies have shown that primary OS mainly metastasizes to the lungs (81.2%), and other metastatic sites include bone and lymph nodes (25). Many researchers have conducted sweeping research on OS metastasis. Sheng et al. (2021) reviewed the metastasis process of OS according to the existing research results and provided a comprehensive understanding of cross-regulatory networks in metastasis based on the diverse biological behaviors and associated mechanistic pathways of OS metastasis (3). Bruland et al. (2005) used a sensitive immunomagnetic detection assay that successfully identified micrometastases from bone marrow and peripheral blood in OS patients (26). Guan et al. (2015) developed a specific molecular probe named CXCR4-targeted near-infrared (NIR) fluorescent imaging agent to detect pulmonary micrometastases in OS cells and mouse xenograft models, with the hope to apply in micrometastasis of human OS (27). Wang et al. (2018) found that ssDNA aptamer LP-16, as a promising molecular probe, can achieve significant targeting efficiency for OS lung-metastasis diagnosis (28).

At present, the primary diagnosis and detection of OS micrometastasis are necessary and important to improve the survival of patients. Further, developing and identifying new therapeutic strategies to combat metastasis is also the key to the treatment of OS.

## Treatment of osteosarcoma

### Traditional treatment of osteosarcoma

Nowadays, despite the development of new therapeutic approaches, the prevailing methods are still surgery, chemotherapy, and radiotherapy. The combination of multiagent chemotherapy with surgical resection or radiation therapy drastically improved the survival rate to become the standard therapy for OS. However, the prognosis of the patients has not improved considerably since then, and limited therapeutic progress of OS treatment has been made for several decades.

Nowadays, the most effective dosage regimen of drugs was still the combination of high-dose methotrexate, doxorubicin, and cisplatin, usually referred to as MAP, similarly to the first agents in the mid-1970s (29). Nevertheless, adding other new chemotherapeutic agents such as ifosfamide and etoposide into the regimen does not improve prognosis effectively (30, 31). Furthermore, problems of drug resistance and side effects, such as cardiac toxicity, nephrotoxicity, and other rare toxicities, have existed subsequently (32). Drug resistance to conventional combination therapies including chemotherapy, as an important obstacle in OS treatment, is closely related to the changes in tumor metabolism (10). Thus, the broader availability of therapeutic techniques and correlative molecular profiling with the biological insights should be further explored in OS treatment.

### Molecular targeted therapy of osteosarcoma

Molecular targeted therapy refers to the utilization of drugs or other substances that target specific molecules to prevent the growth and spread of tumor cells. It is one of the promising methods to further improve the survival rate of patients with OS. Sayles et al. (2019) further extended the non-overlapping whole-genome sequencing (WGS) dataset of OS, which indicated the characterizations of OS genomes consistent with those previously found such as notable gene copy number alterations and multiple structural rearrangements, while six OS subclassifications will be divided by the key gene signaling pathways activated, namely, cyclin E/CDK2, MYC/CDK9, CDK4/CDK6/FOXM1, PTEN/AKT1/PI3K/mTOR, AURKB, and VEGFA/VEGFR, mainly regulating biological processes of the cell cycle, proliferation, cell-cycle progression during G1–S, signaling, mitosis, and signaling angiogenesis, respectively (6). Subsequently, Liu et al. (2021) summarized the preclinical and developmental research of targeted anti-angiogenesis therapy for OS in detail and described the relevant biological mechanism more comprehensively, which further determined the research prospects of targeted therapy, although it was still uncertain in clinical trials (33). Among them, multiple tyrosine kinase inhibitors, such as sorafenib, regorafenib, cabozantinib, lenvatinib, pazopanib, and everolimus, have shown certain efficacy in small sample studies and need to be further confirmed (34). Also, although some drugs targeting the cell cycle and DNA repair like palbociclib (CDK4/6), alisertib (AURKA), and dinaciclib (CDK2) have not been evaluated in clinical trials of OS, those drugs have been proved to induce apoptosis and inhibit cell proliferation *in vitro* or *in vivo* (35).

On the other hand, molecular targeted therapy models of OS included patient-derived xenograft (PDX) and transgenic models, which played an important role in the study of the biological characteristics and clinical trials of human OS (36, 37). However, a number of drawbacks in targeted therapy based on the concept of precision medicine still remain.

Hence, molecular targeted therapy for OS should be predicated on the selection of biomarkers to increase the possibility that a drug is effective in heterogeneous patient populations, rather than defining therapeutic activity through the direct relationship between the target and the drug (30). Therefore, targeted therapy needs further research to evaluate its potential role as a promising novel therapy in the treatment of OS in the future.

### Immunotherapy of osteosarcoma

Studies have shown that OS is a tumor susceptible to immunotherapy, which is largely attributed to the OS cells derived from multipotent mesenchymal stem cells and the changes in the tumor microenvironment (TME) (7, 38). Indeed, more than 100 years ago, surgeon William Coley (1891, 1910) found that the use of a mixture of bacterial toxins to treat patients with bone and soft tissue sarcoma successfully subsided the tumor and first envisaged the immune component of OS treatment (39, 40). In recent years, with the rapid development of immunotherapy, it has been widely used in a variety of malignant tumors. Chen et al. (2021) summarized the current status and breakthroughs of immunotherapy for OS (41). Although the current reports on the clinical immunotherapy of OS are limited, immunotherapy is still considered to be a promising option for the treatment of OS and one of the important approaches to further improve the overall survival rate of patients.

At present, lung metastasis and recurrence of OS are still the main factors limiting the improvement of survival rate. Therefore, activation of associated cells of the TME is one of the therapeutic strategies of OS, tumor-associated macrophages (TAMs), and tumor-infiltrating lymphocytes (TILs) like macrophages and T cells play significant roles in the immune response to OS (42). A variety of immunotherapy methods, such as chimeric antigen receptor T-cell (CAR-T) therapy, immune checkpoint blocking therapy, natural killer (NK) cell infusion therapy, macrophage activation therapy (GcMAF), and oncolytic virus therapy, are constantly explored in the basic research and clinical application of malignant bone tumors. Among them, immune checkpoint inhibitors (ICIS), as a new class of therapeutic drugs, have completely changed the treatment of previously incurable malignant tumors, stimulated interest in this therapeutic method, and opened a new door for the immunotherapy of OS (43).

Besides, although immunotherapy has not yet observed surprising effects in OS clinical treatment, the continuous emergence of newly discovered immune checkpoint blockade targets has created a new dawn for the exploration of OS treatment. Therefore, we believe that unprecedented progress and major breakthroughs will be made in the treatment and understanding of OS in the near future.

## Phototherapy of osteosarcoma

**Figure 1:**
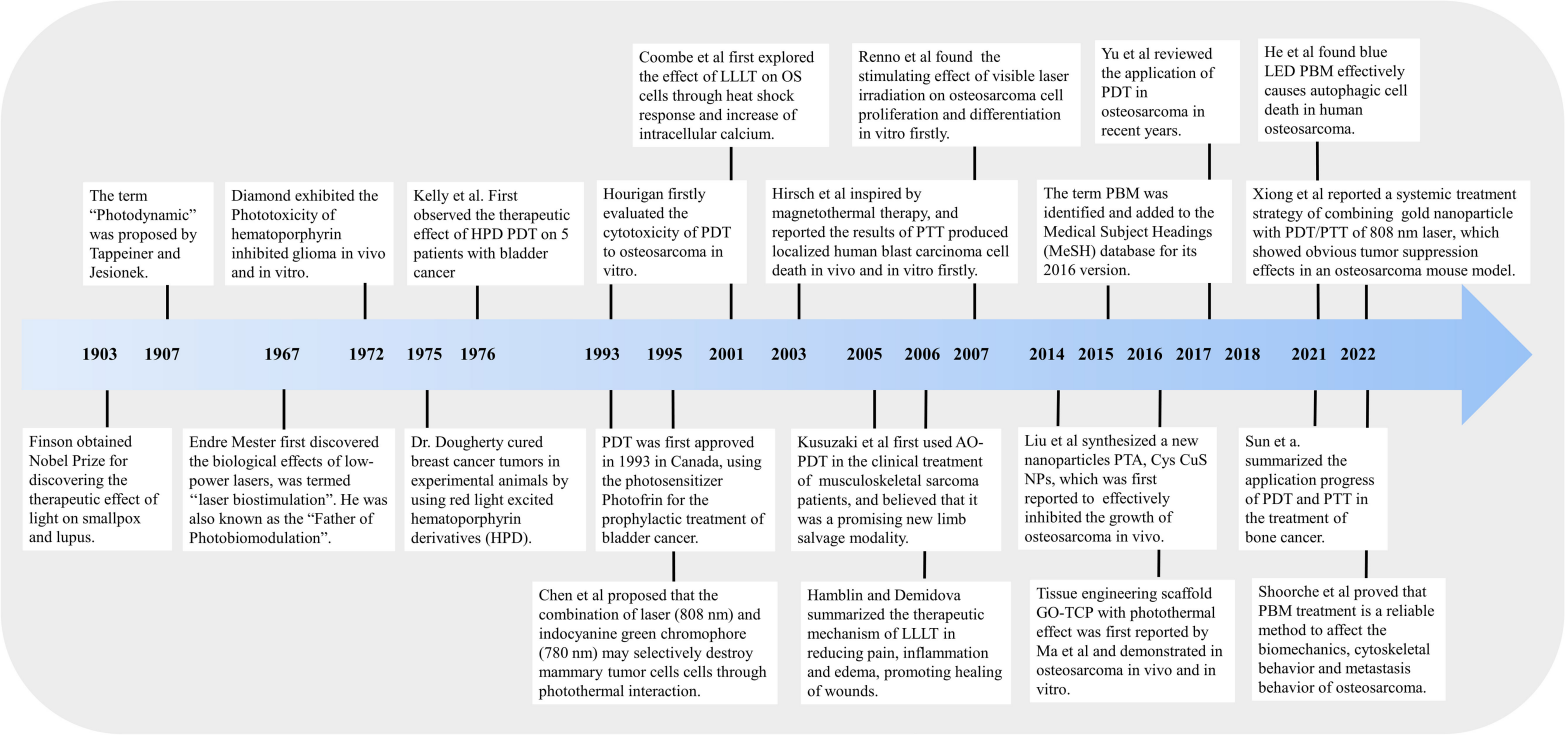
Schematic diagram of research progress of phototherapy (PDT, PTT, and PBMT) in the treatment of osteosarcoma.

**Figure 2:**
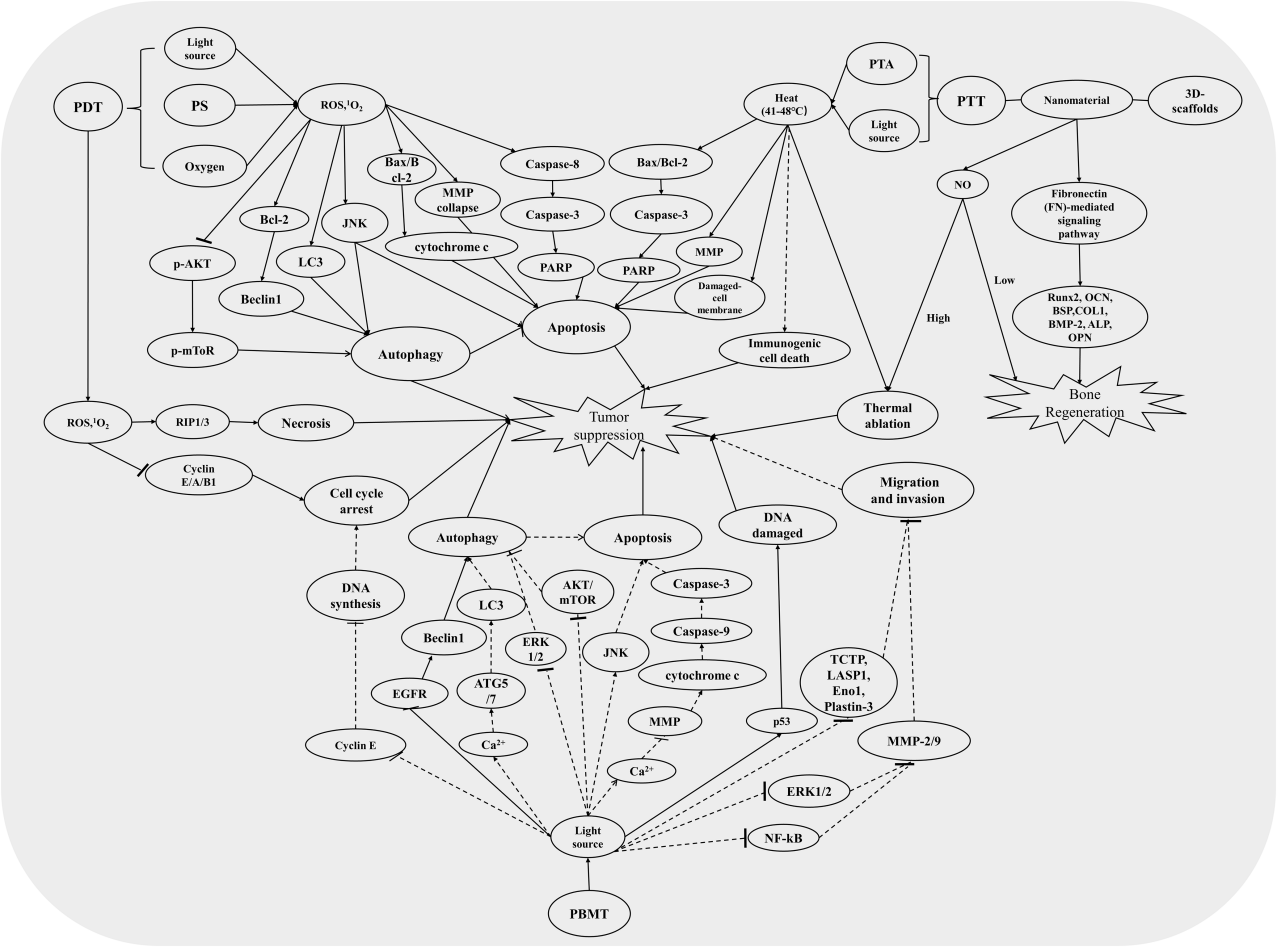
The relevant mechanism pathways involved in PDT, PTT, and PBMT induced an antitumor effect. The solid line represents the pathways that have been studied in the treatment of osteosarcoma, and the dotted line represents the pathways that have been studied in the treatment of other tumors but not in osteosarcoma.

In recent decades, the survival rate of OS has reached a platform period. Although the combination of surgery and other treatments has improved the cure rate of OS to around 70%, the bone defect and complex skeletal rebuilding of the affected limb caused by surgery still limit the application (5). Therefore, more promising novel therapeutic approaches to minimally invasive treatment are receiving more and more attention in tumor-related research. Phototherapy is the clinical application of using light to treat specific diseases, which is safe, non-invasive, economical, and easy to handle, including PDT and PTT (8). Finson obtained a Nobel Prize in Physiology or Medicine in 1903 for discovering the therapeutic effect of light on diseases such as smallpox and lupus. Subsequently, the healing properties to wounds and cytotoxic characteristics for tumor cells of light were gradually identified and widely used in minimally invasive cancer therapy until now (44, 45). On the other hand, PBM, also known as low-level light therapy (LLLT), is a non-thermal and non-invasive therapy that uses light with a specific spectrum (390–1100 nm) to regulate cell life activities and produce therapeutic effects (46, 47). At present, PBM has been widely used to reduce inflammation, relieve pain, and promote wound healing (48). Meanwhile, PBM is also applicated in tumor treatment such as melanoma due to its non-invasive advantages, also named PBMT (10, 11). Accordingly, research of the novel therapies such as PDT, PTT, and PBMT for tumor treatment is extremely important. Here, we have comprehensively summarized the main research progress (**Figure 1**) and related molecular mechanisms (**Figure 2**) of phototherapy in OS.

### Photodynamic therapy of osteosarcoma

PDT, as a non-invasive treatment, has been widely developed and applied in a variety of cancer treatments because of phototoxicity produced by reactive oxygen species (ROS) to kill tumor cells since first approved for clinical applications using the photosensitizer Photofrin for bladder cancer in Canada in 1993 (49). Three basic factors of PDT, namely, photosensitizer (PS), light source, and oxidative stress ( $O_{2}^{-}$ , ROS), were exploited to kill tumor cells. The specific mechanism is that the PSs are activated from the ground state to the excited state under the action of light of a specific wavelength, and the activated PSs are able to directly react with the substrate (such as a cell membrane or molecule) to form free radicals and interact with oxygen to produce ROS (type I reaction) or direct energy transfer to oxygen to form singlet oxygen (^1^O_2_), causing cell damage by ^1^O_2_ directly (type II reaction) (49, 50). In sum, light energy is converted into chemical energy through appropriate PSs in PDT. The selective photodamage and cytotoxicity are important factors in the treatment of different cancers with PDT (51). Also, Dolmans et al. (2003) summarized three main mechanisms of PDT affecting tumor growth, namely, direct cancer cell killing by ROS, vascular damage, and immune response (49).

PS, as one of the important components of PDT, had been summarized in OS treatment by Yu et al. (2017); they found that although PDT had gradually become a hot spot in cancer treatment research, related research in OS was still insufficient, and PSs were limited to more than a dozen types mainly including acridine orange (50, 52). However, the rapid development of nanoparticles (NPs) has provided a major breakthrough for the PS defects of PDT in cancer treatment, including deeper tumors such as OS. The characteristics of modification and enhanced permeability and retention (EPR) effects of NPs reduce the sedimentation and cytotoxicity of PS in the liver, kidney, and other normal organs and tissues and enhance the application prospect of PDT in the treatment of OS recently (53). At the meantime, Yu et al. (2017) summarized seven feasible improvements in OS PDT, including the further exploration of PSs and light sources, and the combination with other treatment technologies such as targeted therapy and immunotherapy (50). Therefore, further study of PSs will contribute to the prominent development and application of PDT in the treatment of OS.

Furthermore, PS could target cancer cells and destroy them with the excitation of appropriate wavelength and light intensity (54). As early as 1993, Hourigan et al. firstly evaluated the cytotoxicity of PDT to OS *in vitro*. They found that the cell viability of human osteosarcoma cells (HOS) was inversely proportional to the energy dose (Dose = Irradiance × Time) after incubation with Photofrin at 3 μg/ml for 72 h followed by light exposure (630 nm, 600 mW). The cell viability is less than 50% when the energy dose was greater than 7 J/cm^2^, effectively inhibiting the proliferation of OS cells (55). Later, Kusuzaki et al. (2005) applied photodynamic therapy using acridine orange (1 μg/ml) (AO-PDT) to the clinical treatment of 10 musculoskeletal sarcomas patients, only one patient had local tumor recurrence, and the limb function in other patients returned to normal without other complications. They believed that AO-PDT had an obvious inhibitory effect on the local recurrence and was a promising novel therapy for patients of musculoskeletal sarcoma (56). Subsequently, a number of PSs such as 5-methylene blue (57, 58), aminolevulinic acid (ALA) (59, 60), HiPorfin (61), 5,15-bis(2-bromo-5-hydroxyphenyl) porphyrin (62), aloe-emodin (60), Foscan and Foslip (63), pyropheophorbide-α methyl ester (MPPa) (64), and zinc phthalocyanine-bovine serum albumin (ZnPc/BSA) nanoparticle (65), were widely applied in scientific research and clinical treatment of OS. Substantial research about light wavelength mainly focused on 600–800 nm; the NIR spectral region can effectively affect the deeper tissues (66). Also, with the rapid improvements of lighting technology, LED gradually became common in OS PDT due to its superior abilities to control light parameters. For example, White et al. (2016) found that the OS MG-63 had the highest cell death rate under 1 mM ALA after illumination with 3 J/cm^2^ LED light (636 nm) (67). Another study conducted by Tu et al. (2016) also found notable toxicity in MG-63 human osteosarcoma cells at 10 µM aloe-emodin and 4.8 J/cm^2^ LED light irradiation conditions (430 nm) (60). Also, Yu et al. synthesized ZnPc/BSA nanoparticle as a specific PS to evaluate the antitumor effects of ZnPc/BSA-induced PDT on OS under LED illumination (660 nm, 0.5 W/cm^2^, 1.8 kJ/cm^2^) (65).

Besides, ROS plays an extremely important role in PDT on cancer treatments. In Sun’s research, they assessed the ROS level using carboxy-H2DCFDA as an ROS indicator. They found a remarkable increase of ROS in OS cells treated with HiPorfin-PDT and that the expression levels of proteins (Bcl-2, Bax, cleaved-caspase-3, cleaved PARP-1) related to apoptosis and cycle arrest were significantly increased (61). Another study showed that AE-PDT effectively reduced the viability of MG-63 cells and induced autophagy and apoptosis by activating the ROS-JNK signaling pathway (60). Besides, a wealth of studies indicated that the PDT on OS treatment also concentrates on ROS-induced cell death; the relevant pathways, such as apoptosis and necrosis by cell-cycle arrest, vessel injury, autophagy, and immune response, had been concluded (50, 68). We had summarized the mainly molecular mechanism of PDT in the treatment of OS in **Figure 2**. Nevertheless, although the depth of OS is less than that of glioma and pancreatic cancer, as a deeper tumor (shallower than other deep tumors), due to the coverage of muscle and skin, there will be insufficient light and surface tissue damage in PDT (69). Recently, two-photon photodynamic therapy has been gradually studied and applied in malignant tumors, including osteosarcoma, because of its deeper tissue penetration and oxygen independent characteristics (70, 71). Dobos and coworkers (2019) explored the efficiency of two-photon excited PDT (TPE-PDT) in a 3D osteosarcoma model; results showed a considerable decrease in cell viability after TPE-PDT (72). Nonetheless, there are still limitations like the lack of efficient and safe two-photon PSs and difficulties in practical application of double pulsed laser sources. Research on PDT in OS is still in the tumor model experiments, and its clinical application is still very limited. Thus, more comprehensive and in-depth research should be explored in the field of PDT.

### Photothermal therapy of osteosarcoma

PTT is also a minimally invasive, controllable, and highly effective antitumor therapeutic modality (73). In early 1995, Chen et al. firstly found that indocyanine green and 808-nm diode laser combined to produce a photothermal effect could kill breast tumor cells (74). Subsequently, the utilization of PTT for cancer therapy was first reported by Hirsch et al. in 2003, which was inspired by magnetothermal therapy (75). In 2014, Liu et al. synthesized a new nanoparticle PTA, Cys CuS NPs, which was first reported to effectively inhibit the growth of osteosarcoma *in vivo* (76). Then, a large number of studies have been published on the application of PTT on OS *in vivo* and *in vitro* since 2014. Here, we present key findings in **Figure 1**.

Two key elements in PTT, namely, photothermal agent (PTA) and light source, were exploited to kill tumor cells. At present, four main types of PTAs, namely, metal, carbon, semiconductor, and organic molecule-based materials, were widely investigated in PTT (8). However, sometimes heat would inevitably leak out of the target tissue and damage the surrounding normal tissue due to the limitations of PTAs previously. Therefore, with the dramatic progress of PTAs by modification and nanoformulation, some nanomaterials, as the most promising PTAs with continuous in-depth research nowadays, were gradually used as effective photothermal sensors because of their unique properties and special chemical structures, which could effectively generate heat in the target cancer tissue through the optical absorption, instead of the healthy tissues (73). Additionally, a specific light wavelength, mainly including NIR light, will be absorbed by PTAs to efficiently convert to heat by local irradiation of the tumor regions. As tumor cells are more sensitive to heat-induced cytotoxicity, when the local tumor area temperature increases to the range of 41°C–48°C owing to high photothermal conversion performance, heat induced cell biological processes and caused cancer cell death (77). Recently, PTT with other therapies, such as chemotherapy and immunotherapy, was able to more comprehensively and efficiently improve the treatment efficacy of different cancers (50).

At present, the majority of studies on PTT treatment of OS mainly focus on the synergistic treatment of nanomaterials, modifying substances or other chemotherapy-related drugs (8). Among them, gold nanoparticles (AuNPs) are extensively studied in OS PTT because of the higher NIR absorption coefficient and stability compared with other nanomaterials. Liu et al. (2017) first designed a gold nanoshell-coated betulinic acid liposome (AuNS-BA-Lip) delivery system, which strongly absorbed the 808-nm NIR laser (2 W/cm^2^, 10 min) and showed the pronounced antitumor effects in 143B cells and corresponding tumor-bearing mice (78). Recently, a systemic treatment strategy of a gold nanoparticle combined with PDT/PTT by 808-nm laser showed obvious tumor suppression effects in an osteosarcoma mouse model (79). Also, Sun et al. (2021) comprehensively summarized the progress of PTT and PDT in bone cancer (8). On the other hand, some nanoparticle-integrated 3D-printing scaffolds with PTT played an important role in OS treatment and NO-associated bone regeneration (80). Therefore, PTT, as a novel and practicable therapeutic approach, will also become a promising diagnosis and therapeutic method for OS.

Also, the molecular mechanisms related to the treatment of OS by PTT have also been studied, although they have not been fully elucidated. Studies have shown that the inhibitory effect of PTT on OS is related to apoptosis (81–83) and thermal ablation pathways (84, 85). Flow cytometry is widely used in apoptosis analysis. Lee and colleagues (2021) demonstrated that apoptotic cells were more than necrotic cells on the PLIN (indocyanine green and diethylenetriamine/nitric oxide adduct-loaded polylactic acid) monoliths after laser irradiation (808 nm, 0.7 W/cm^2^, 5 min) in OS MG63 cells; the expression levels of related proteins, such as cleaved PARP and cleaved caspase-3, were remarkably increased (81). Dang et al. (2021) had similar results *in vitro*, and further study in mice found that a special scaffold had a remarkable antitumor effect on OS mice after NIR laser irradiation (808 nm, 0.9 W/cm^2^, 10 min) and no obvious damage in mice’s other organs (82). Some researchers also discovered that a multifunctional biomaterial system combined with PTT plays a significant role in OS therapy and bone regeneration (64, 85). Moreover, Dong et al. (2020) reported a multifunctional bioceramic platform with carbon aerogel (CA), which plays an efficient therapeutic effect in OS through thermal ablation and promotes bone regeneration *via* the fibronectin-mediated signaling pathway; the expression levels of a substantial number of osteogenic genes such as ALP, BMP2, OCN, and OPN are dramatically upregulated (84). Here, we summarized the molecular mechanisms of PTT inhibition of OS related to most of the findings, as shown in **Figure 2**.

### Photobiomodulation therapy of osteosarcoma

PBMT is one of the exciting hotspots in the current biomedical research field. Actually, as early as more than 50 years ago, surgeon Endre Mester, known as “father of photobioinduction,” first discovered the biological effects of low-dose laser light and recorded the experiment of the Arndt-Schultz biophysical law of laser light (86). Therefore, this treatment method had been called low-level laser therapy (LLLT). Until 2015, the more precise and comprehensive term PBM was identified and added to the Medical Subject Headings (MeSH) database for its 2016 version (46). Also, in addition to the laser as the light source, other new types of optical devices such as LED and broadband light sources are gradually applied in the field of treatment.

At present, most studies indicate that PBMT plays an important role in the aspects of wound healing (87), anti-inflammatory (88), traumatic brain injury (89), and related clinical treatment applications (48). In addition, PBMT is gradually emerging in oncology applications and its prognosis. A substantial number of studies showed that PBMT had a significant inhibitory effect on melanoma cells under suitable light parameters. At the same time, the related molecular mechanisms have also been deeply studied. Ohara et al. (2002) found that 470-nm LED blue light inhibited the proliferation of melanoma cells associated with cell-cycle arrest (90). Oh et al. (2015) observed that blue light can further induce cell apoptosis and inhibit the proliferation of melanoma B16-F10 cells by activating the mitochondria (10). Chen et al. (2022) summarized the effect of different light parameters on the growth of melanoma and its molecular mechanism; they believed that the inhibitory effect of PBMT is related to the influence of OPN photoacceptors and the activation of mitochondria and some signaling pathways (11). A similar result was evaluated by Shakibaiea et al. (2020), using breast cancer cells (MCF-7) with inhibition of cell proliferation and metabolic activation by 435-nm LED irradiation (91). Besides, the authors also found that radiation with a wavelength of 629 nm induces enhanced metabolic activity including the expression of lactate dehydrogenase A and glutaminase (91). Meanwhile, Kara et al. (2018) showed that LLLT irradiation (1,064 nm, 100 mJ) promoted the proliferation of osteosarcoma cells (Saos2) and lung carcinoma cells (A549) (92). In addition, Zhang et al. (2008) reported that low-power laser irradiation (<50 J/cm^2^) by He–Ne laser (632.8 nm) promoted Hela cell viability by induced ROS-mediated Src activation (93). Also, Tam et al. (2020) raised the question that normal cells and tumor cells have different responses to LLLT (94). In sum, the controversial results on cancer cells are associated with various factors; wavelength is one of the main influencing factors. A substantial number of studies showed that blue light PBMT played a significant role in inhibiting cancer cell proliferation (90, 91, 95). Therefore, OS, as one of the malignant bone tumors, had been found to have a stimulating effect of infrared laser irradiation as early as 2001 (96). Although it had no significant effect on cell proliferation, heat shock response and the increase of intracellular calcium had been found in OS cells (96). Subsequently, Renno et al. (2006) found that visible light (670 nm) also had a stimulating effect on OS cells (97). Recently, Feng et al. (2019) reported the results on the combination of blue LED irradiation and ATO suppressed cell proliferation, increased apoptosis, inhibited cell migration and invasion, and found that it was related to ROS accumulation and DNA damage-mediated p53 activation (95). Besides, He et al. (2021) found that blue LED PBMT effectively causes autophagic cell death in human OS cells, which was induced by promoting ROS and the EGFR/Beclin-1-mediated signaling pathway (98). Recently, Shoorche et al. (2022) also discovered that red laser irradiation (650 nm, 780 nm) inhibited OS cell migration and provided a quantitative description of cytoskeleton changes through a structure of F−actin analysis (99). Therefore, PBMT has great research value and prospects in the treatment of OS. More key research progresses and related molecular mechanisms on PBMT are shown in **Figures 1**, **2**.

In recent years, increasing evidence has shown that different light parameters, including wavelength, irradiance, and energy densities, have different effects among different tumor types including OS, promoting or inhibiting proliferation (100). However, with the rapid development of LED technology, the preferred light source has been gradually developed in the treatment of a variety of PBMT diseases because of their lower cost, high variability of wavelengths, safety of device, and wearability compared with laser devices (13, 101). Hence, blue LED light PBMT will become one of the most promising approaches for the treatment of OS.

## Blue light photobiomodulation therapy in cancer treatment

### The biological function of blue light in osteosarcoma and other cancers

Several studies have indicated that light irradiation (LED or laser) can trigger various physiological processes by affecting human skin, which is pretty significant for human health (102, 103). In the current studies, we have found that the exploration of ultraviolet and far-infrared light has been deeply analyzed, and visible light has also been found to have various biological functions (46). Blue light (400–500 nm) LED irradiation is known to have many promising effects and can be used in different treatment modalities. Although the role of blue light irradiation in mammalian cellular molecular processes and its application in phototherapy applications is currently poorly understood, this emerging field is receiving increasing attention.

**Figure 3:**
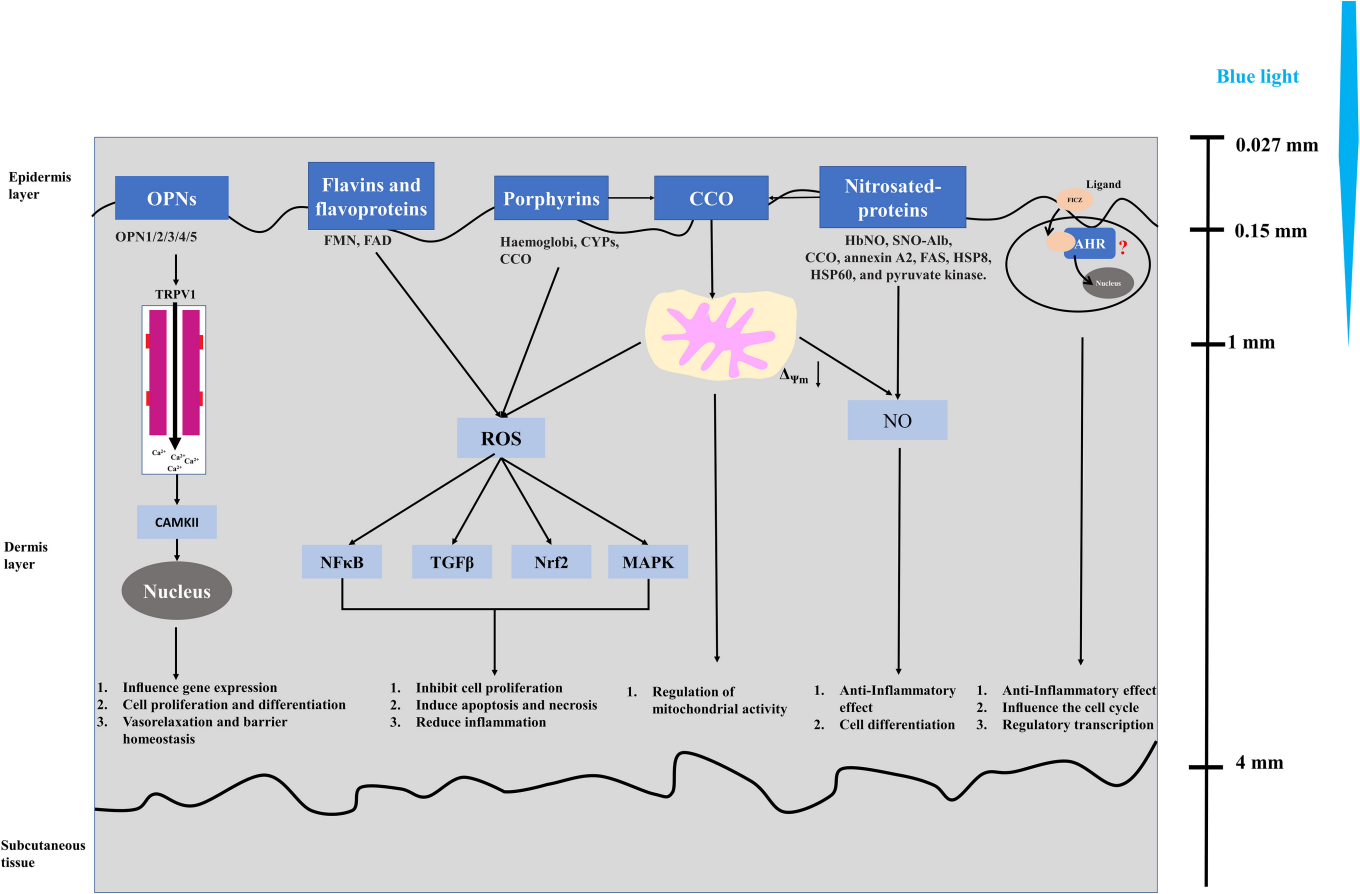
Mechanism of blue light affecting non-pigmented mammalian cells.

Blue light triggers signal cascades and corresponding cellular reactions through a molecular photoreceptor or an endogenous photosensitizer to affect some biological systems. Current research indicates that photoreceptors of blue light in plants and bacteria have relatively mature research results. For example, cryptochromes have obvious light-dependent functions, but there are still disputes on cryptochromes as a photoreceptor in mammals (104, 105). Despite the controversy in illustrating relevant photoacceptors (chromophores) of blue light in mammal cells, five different photoacceptors, namely, flavins (106–108), porphyrins (109, 110), nitrosated proteins (111, 112), opsins (OPN) (113–115), and cytochrome c oxidase (CCO) (116–118), have been identified in non-pigmented mammalian cells (keratinocytes and fibroblasts), while previous studies mainly focused on pigmented cells, such as retinal ganglion cells (119, 120). Blue light stimulates OPN to cause structural changes and induce the activity of the downstream transient receptor protein (TRP) channel (TRPV1), then calcium influx activates CaMKII, which affects the gene transcription level in the nucleus and further affects cell promotion and differentiation, vasorelaxation, and barrier homeostasis (113–115). Flavin (460 nm) and porphyrin (410–440 nm) absorb blue light to produce ROS and activate NFκB, TGFβ, Nrf2, and MAPK signaling pathway and further inhibit cell proliferation, induce apoptosis and necrosis, and reduce inflammation (106–110). Nitrosated proteins (420–490 nm) induce the non-enzymatic release of bioactive compounds in the blue spectrum and play an important role in anti-inflammatory effect and cell differentiation (111, 112, 114). The PBM effect of CCO mainly affects the mitochondrial activity through the mitochondrial electron transport chain and then affects the biological mechanism of cells (116, 117). On the other hand, CCO contain porphyrin and also have the function of nitrite reductase to produce NO in mitochondria; it is considered as another possible mechanism of CCO blue light response (114, 118). Moreover, Becker et al. (2016) found that aryl hydrocarbon receptor (AHR) may be a possible target for blue light PBM (121). Moreover, 6-formylindolo[3,2-b] carbazole (FICZ), as a photoinduced ligand of the aryl hydrocarbon receptor (AHR), is a tryptophan photoproduct, which has been widely studied (122). These studies on blue light receptors play an important role in exploring biological mechanisms. Therefore, we briefly summarized the current main research on blue light receptors and their related biological mechanisms as shown in **Figure 3**.

Considerable research showed that phototherapy based on blue light has produced beneficial consequences such as treatment of neonatal jaundice (123–125), regulation of melatonin and circadian rhythm (126–128), anti-inflammation (129–131), immunomodulation (132), wound healing (133–135), tissue regeneration, and anticancer therapies (103, 114, 133, 136). Furthermore, blue light reduced the follicular colonization of *Propionibacterium acnes* by activating the endogenous bacterial porphyrin and inhibiting acne development, while possibly interfering with lipogenesis in adipocytes and suppressing sebum formation for acne treatment (137). Blue light irradiation (450 nm) induced the release of nitric oxide (NO) from cytochrome oxidase and other mitochondrial heme proteins but can reverse the effects of NO and improve mitochondrial function, while the absorption of light by hemoglobin can cause local temperature increase, promoting wound healing through coagulation effect (138, 139). Kim et al. (2016) demonstrated that the anti-inflammatory effect of blue light (410 nm) on human epithelial keratinocytes (NHEKs) was associated with NO and S-nitrosylation (140). Also, blue light irradiation (420 nm) affected the proliferation, differentiation, and growth of human dermal fibroblasts and even had dose-related toxic effects (141). Becker et al. (2016) discovered that the proliferation of immortalized human keratinocytes (HaCaT) induced by blue light showed a biphasic dose–response curve, similarly to the PBM conclusion proposed by Hamblin et al. (121). Furthermore, Oh et al. repeatedly found that blue light could even reduce cell viability and induce apoptosis of different types of cancer cells (10, 13, 14). Those findings determined the effect of blue light on the proliferation, differentiation, apoptosis, inflammation, vasorelaxation, and barrier homeostasis of cells, while others describe cytotoxicity (142). Despite controversy over the role of blue light, these results and conclusions still play a significant role in the exploration of the physiological role of blue light, also laying a biological foundation for the clinical application of blue light in phototherapy.

However, if the light parameters are not appropriate, blue light will also be harmful to cells, which is described as cytotoxicity. Relevant studies have shown that blue light with high dose/irradiance may cause damage to keratinocytes, fibroblasts, retinal epithelial cells, skin-derived endothelial cells, and other mammalian cells in addition to pigmented cells (143). Consequently, the biological effects of LED blue light irradiation are dose-dependent; it is necessary to define parameters like wavelength, dose (fluence, energy density), irradiance (intensity, power density), light mode (continuous wave, pulsed wave (peak irradiance, duty cycle)), treatment interval, light source type, and cellular species so as to obtain specifically the cellular responses and better clinical treatment effect of cancers in the future (143).

### Application of blue light PBMT in different cancers

Blue light, as a visible light with a wavelength range of 400–500 nm, has been clinically applied successfully comprising neonatal jaundice, psoriasis (Pv), atopic dermatitis (AD), eczema, acne, and other inflammatory skin conditions, although the number of available clinical studies evaluating the efficacy of blue light treatment is still limited (103, 136). Furthermore, recent studies have demonstrated that blue light irradiation inhibits the proliferation of multiple types of cancer cells *in vitro* and *in vivo*, including colon cancer cells (14, 144, 145), malignant glioma cells (12), melanoma cells (10, 146, 147), B-cell lymphoma cells (13), fibrosarcoma cells (14), pancreatic cancer cells (15), cutaneous squamous cell carcinoma cells (CSCC), epidermoid carcinoma cells (148, 149), leukemia cells (Kasumi-1) (150), bladder cancer cells (151), colorectal cancer cells (152, 153), breast cancer cells (91), and human OS cells (95, 98). Here, we have briefly summarized the relevant research of blue light PBMT in the field of cancer treatment (**Table 1**).

**Table 1:** Summary of the most relevant experimental studies on cancers treatment by blue light irradiation.

Year	Cancer cells	Wavelength (nm)	Irradiance (mW/cm^2^)	Irradiation time	Dose (J/cm^2^)	Outcomes	Molecular mechanism	Reference
2013	Malignant glioma cells (U87 and H4)	460-485	*In vitro*:164; *In vivo*:170 (Subcutaneous glioma model); 1000(Intracranial glioma model)	*In vitro*: 1 h (10 Hz, 50 ms); *In vivo*: 30 min (2 weeks)	No information	Appropriate parameters of blue light precisely inhibited proliferation and increased death of glioma cells h *in vitro* and *in vivo* by expressing the engineered opsin gene ChETA.	Light-induced the membrane depolarization and Ca2+ channels change mediated glioma cell death associated with cell cycle arrest, mitochondria-mediated apoptosis.	(12)
2014	Epidermoid carcinoma cells (A431)	400-500	*in vitro*: 500	30 s, 90 s	*In vitro*: 15, 45; *In vivo*: 45	Blue light treatment reduced viability of epidermoid carcinoma cells in a dosage-dependent manner, and decreased tumor cell growth, proliferation, and oxidation levels in xenograft model.	Blue light activated the phase 2 response (glutathione, the peroxiredoxins, and heme oxygenase 1), suppressed mitochondrial function.	(149)
2014	Colon cancer cells (HT29 or HCT116)	465	15 or 30 mW	10 min/day, 5 days	No information	Blue LED irradiation inhibited the proliferation and promoted apoptosis of colon cancer cells.	The inhibitory effect of blue light on lymphoma cells was associated with cell cycle arrest, inhibition of autophagy and ERK pathway, and activation of external apoptosis pathway and JNK pathway.	(145)
2015	Melanoma cells (B16-F10)	450	*In vitro*: 15.6; *In vivo*: 0.6.	*In vitro*: 1, 2, 3, 4 h; *In vivo*: 3 h blue LED on and 5 h blue LED off as one cycle, a total of 39 cycles	No information	Blue light had anti-proliferative and pro-apoptotic effects on melanoma cells, as well as reduce tumor growth.	The inhibitory effects were related to mitochondrial membrane potential, cell cycle arrest, caspase-3 activation, and ROS production.	(10)
2016	Mouse B-cell lymphoma cells (A20 and RAMOS)	450	*In vitro*: 6.3; *In vivo*: 4.1	*In vitro*: 1, 2, 3, 4 h; *In vivo*: 3 h/days, 3 days.	No information	Blue LED inhibited cell growth and triggers apoptosis by induction of autophagy in lymphoma cells, while the survival rate of leukemia mice was improved.	The anti-tumorigenic effects were associated with formation of autophagosomes, intracellular ROS production, mitochondrial membrane potential, and caspase-3 activation.	(13)
2017	Mouse colon cancer (CT-26); Human fibrosarcoma cells (HT-1080)	450	*In vitro*: 6.3; *In vivo*: 8.23	*In vitro*: 30, 60 min; *In vivo*: 3 h/days, 3 days	No information	Blue LED irradiation inhibited the migration and invasion of solid tumor cells and decreased cellular proliferation of CT-26 and HT-1080 cells.	The anti-metastatic and anti-proliferative effects were associated with MMP-2, MMP-9, and P38 MAPK phosphorylation.	(14)
2018	Human colon cancer cells (HT-29 or HCT-116)	465	30	30 min	No information	Blue light reduced cell viability and suppressed the growth of colon cancer cells.	Autophagy Opn3 photoreceptor pathway could be the mechanism of cell growth inhibition.	(144)
2018	Colorectal cancer cells (SW620 and HT29)	470	20	No information	0, 72, 144, 216, and 288	Blue LED irradiation inhibited the growth, proliferation, migration, and EMT process of CRC cells, and induced apoptosis.	The inhibited effects may be related to increased ROS accumulation and DNA damage.	(152)
2019	Human bladder cancer cells (5763)	400-500	No information	Every 24 h for 120 h culture time	8.7, 17.5	Combination therapy of blue light irradiation and chemotherapy inhibited the bladder cancer cell viability in a dose-dependent manner and attenuated drug resistance.	Synergistic effect inhibited cell proliferation, diminished glucose consumption and lactate formation, and arrested cell cycle	(151)
2019	Osteosarcoma cells (U-2 OS)	470	100	30 min	180	The combination of blue LED irradiation and ATO suppressed cell proliferation, increased apoptotic, inhibited cell migration and invasion.	The synergistical antitumor effects were associated with accumulation of ROS, DNA damage, and p53 activation.	(95)
2020	Melanoma cells (G361 and A375)	460	8.4	Dark and blue light: 1 s:30 s	No information	This study provided a light-induced CRISPR-Cas9 system, inhibited the progression of melanoma cells.	The inhibitory effect of this blue light-induced system was associated with mutant gene BRAF V600E, restrained cell invasion, inhibited the migration, and promoted cell apoptosis.	(146)
2020	Human pancreatic cancer cells (PaCa-2, PANC-1, and BxPC-3)	460	*In vitro*: 5, 10; *In vivo*: 0	*In vitro*: 3 or 5 h/day for 5 days; *In vivo*: 2 h/day for 5 weeks	*In vitro*: 12; *In vivo*: no information	Blue LED irradiation inhibited proliferation of pancreatic cancer cells and tumor growth, but it did not affect normal pancreatic cells.	Blue light promoted apoptosis by arresting the G0/G1 cell cycle and regulating the related proteins of apoptosis (PARP, BAX and Bcl-2, p53) and AKT/mTOR signaling	(15)
2020	Colorectal cancer cells (HCT116 and HT29)	465	3 × 10^4^ lux	1, 2, 3, 4, 5, and 6 h	No information	The combination of blue LED irradiation and two anticancer drugs (AT406 and rocaglamide) causes apoptosis of colorectal cancer cells and had a stronger anticancer effect.	The anticancer effect of blue LED combined with drugs played a role by influencing the genes expression of apoptosis, autophagy, and proliferation, as well as ROS production.	(153)
2020	Breast cancer cells (MCF7)	435	No information	Every 24 h for 96 h culture time	17.5	Blue light irradiation diminished proliferation and inhibited metabolic activation of MCF-7 cells	The cytotoxicity effect of blue irradiation inhibited glycolytic pathway and glucose consumption, and lactate formation was decreased.	(91)
2021	Melanoma cells (B16F10)	457	Average irradiance: 0.19; Peak irradiance: 0.95	Time: 100 min; Frequency (Hz): 4 kHz Duty cycle: 20%	1.14	The effect of pulsed PBM on melanoma is proposed for the first time. It is suggested that the pulse PBM significantly inhibit cell viability and cell necrosis than CW group, including anti proliferation and cell necrosis.	The significant inhibitory effect of pulse-PBM induced by activating Caspase-3, causing parallel damage to mitochondria and lysosomes.	(147)
2021	Epidermoid carcinoma cells (A431)	465	0.84	12 h/day, 3 days	123	The combined treatment of blue light and cis-platinum was more effective in reducing cell viability and increasing apoptotic of cells	The cytotoxic effects of combination were related to S and G2/M cell cycle arrest and the expression of necroptosis−related proteins (Casp−9, Casp−8, Casp−3, Bid, Bax, Cyt c, p53, Aif) and apoptotic pathway.	(148)
2021	Osteosarcoma cells (U-2OS, 143B)	470	100	30 min, 1 h, 2 h, 3 h	180, 360, 720, 1080	Blue LED irradiation significantly suppressed the proliferation, migration and invasion of OS cells.	The antitumor effects of blue light on OS by triggering ROS and EGFR/Beclin-1-mediated autophagy signaling pathway.	(98)
2022	Leukemia cells (Kasumi-1)	456	No information	2 h	No information	Blue light irradiation decreased the proliferation and promoted the apoptosis ratio of the Kasumi-1.	The high suppression efficiency was triggered by activating ROS, decreasing MMP, increasing expression level of caspase-3/9 and attenuating AML1-ETO.	(150)

MMP2, matrix metalloproteinase 2; CRC, colorectal cancer cells; EMT, epithelial–mesenchymal transition; ATO, arsenic trioxide; PBM, photobiomodulation; Mtus1, tumor-suppressor gene 1 associated with microtubules; CSCC, cutaneous squamous cell carcinoma; MMP, mitochondrial membrane potential; ROS, reactive oxygen species; AML1-ETO, the chromosome translocation generates the fusion oncoprotein.

The relationship between blue light and bone metabolism has become the focus of attention in the field of orthopedics recently. Blue light stimulation (480 nm) induced an increase in tartrate-resistant acid phosphatase activity of RAW264.7 cells while promoting osteoclast differentiation (154). Yuan et al. (2017) also found that blue light (470 nm) inhibited the proliferation of bone marrow-derived mesenchymal stem cells and osteogenic differentiation but promoted apoptosis (142). Consequently, we inferred that blue LED light irradiation showed significantly suppressed effects in multiple cancer cell lines including cells associated with bone metabolism. Although the mechanisms leading to cell death were not identical in each cell line analyzed, it was reasonable to believe that blue light irradiation was one of the important and effective anticancer therapies in the clinical treatment of future.

At present, a number of studies and clinical treatments have shown that OS therapy still have some limitations such as multidrug resistance, high cost, and the initial stage of the therapeutic methods (10, 32, 33). Lately, blue LED irradiation-based phototherapy has gradually emerged as a new approach to the treatment of cancers. Although the efficacy of blue light LED therapy in the treatment of OS has not been clarified, some studies have shown that blue light also has an inhibitory effect on OS cells. Feng et al. (2019) observed that the combination treatments of blue LED irradiation (470 nm) and antitumor agent-arsenic trioxide exert synergistical antitumor effects in OS cells, and the synergistic manner suppressed cell proliferation, migration, invasion, and induced apoptosis of OS cells (95). Simultaneously, they proposed relevant biological mechanisms related to ROS accumulation, DNA damage, and *p53* activation (95). Additionally, He et al. (2021) evaluated the antitumor effects of LED blue light therapy individually on human OS cells for the first time, and the results showed that blue LED irradiation significantly inhibited the proliferation, migration, and invasion of human OS cells by triggering ROS, inducing cell apoptosis and autophagy pathways, providing a potential approach and strategy for human OS treatment (98). Also, the investigators found that circadian rhythm may be closely related to endogenous control of tumor progression, including pancreatic adenocarcinoma (155), Glasgow osteosarcoma (155), breast cancer (156), and colorectal tumor (157). We know that light is one of the important factors affecting circadian rhythm. *Cry1* and *Cry2* (cryptochrome circadian clock) are the important core clock genes that achieve tumor suppression by affecting the cell cycle (158). Therefore, we inferred that *Cry1* and *Cry2* will play an essential role in the process of OS suppression by blue LED light.

Also, this laboratory found that pulsed blue light could significantly inhibit OS cell line MG63, which was related to SOCS3 (suppressor of cytokine signaling 3) protein (not shown). Therefore, we speculated that a difference in the inhibitory mechanism of pulsed light and continuous wave light on OS cells may exist. Also, the phenotype of blue light inhibiting OS cells is consistent with the results we summarized, showing that blue LED irradiation suppressed proliferation in various cancer cells (**Table 1**).

Although some cell experiments have basically verified that blue light has an inhibitory effect on OS, relevant clinical treatments have not been reported, so the clinical application of blue light LED irradiation for OS still faces great challenges. Hence, it is necessary to further strengthen the research of blue light therapy in the treatment of OS *in vivo* and *in vitro*, and the relevant molecular and cellular mechanisms still need to be further explored.

## Conclusion

Here, we comprehensively summarized the therapeutic advances of OS, reviewed the current situation of traditional treatment methods of OS, and prospected the research progress of new non-invasive phototherapy on the prognosis of OS. Among them, blue photobiomodulation therapy (PBMT) has been paid more attention to in cancer treatment because of its simple operation and effective results currently. A substantial number of studies demonstrated that blue light effectively inhibited tumor proliferation. Although blue continuous wave light PBMT in different cancer cell lines and superficial skin tumors had made some research progress, it still faces great limitations in deep tumor treatment. Inhibition of tumor growth by blue LED light may also be related to the effects of SOCS3 and CRY1/2, which needs further verification. In sum, in-depth studies of a series of treatment methods and related tumor killing mechanisms are expected to break the status quo, stating that the survival rate of osteosarcoma has reached the platform in recent decades and further improved the cure rate and prognosis of patients with OS.

## Author contributions

ML and YL contributed to the design of the review and checked and revised the manuscript. JY collected information and wrote the main manuscript. QF and HJ edited the manuscript. ML and YL served as the authors responsible for contact and ensuring communication. All authors contributed to the article and approved the submitted version.

## Funding

This work was supported by grants from the National Natural Science Foundation of China (No. 81671376).

## Conflict of interest

The authors declare that the research was conducted in the absence of any commercial or financial relationships that could be construed as a potential conflict of interest.

## Publisher’s note

All claims expressed in this article are solely those of the authors and do not necessarily represent those of their affiliated organizations, or those of the publisher, the editors and the reviewers. Any product that may be evaluated in this article, or claim that may be made by its manufacturer, is not guaranteed or endorsed by the publisher.
